# Evaluation of multi-sample 16S ribosomal DNA sequencing for the diagnosis of postoperative bone and joint infections during antimicrobial treatment

**DOI:** 10.1186/s13104-022-05992-7

**Published:** 2022-03-22

**Authors:** Katja Wallander, Martin Vondracek, Christian G. Giske

**Affiliations:** 1 Department of Infectious Diseases and Venhälsan, Södersjukhuset, Stockholm, Sweden; 2 Division of Clinical Science and Education, Södersjukhuset, Karolinska Institutet, Stockholm, Sweden; 3 Department of Clinical Microbiology, Karolinska University Hospital Stockholm, Stockholm, Sweden; 4 Division of Clinical Microbiology, Department of Laboratory Medicine, Karolinska Institutet, Stockholm, Sweden

**Keywords:** DNA sequencing, Arthritis bacterial, Osteomyelitis, Coinfection, Prosthesis-related infection

## Abstract

**Objectives:**

Clinicians worldwide struggle to identify the bacterial aetiology of bone and joint infections. Failure to unequivocally identify the pathogen is linked to poor clinical outcomes. We explored the added value of analysing multiple samples per patient with 16S ribosomal DNA (16S rDNA) sequencing in diagnosing postoperative bone and joint infections. All patients had received antimicrobials prior to sampling, and false-negative cultures could be suspected. Bone biopsies obtained from patients with postoperative bone and joint infections for cultures were also subjected to 16S rDNA sequencing.

**Results:**

In 5/28 infectious episodes, sequencing identified the causative organism of the infection when cultures failed. In 8/28 episodes, the methods led to different results, potentially leading to different antimicrobial choices. The analysis of multiple samples per patient helped rule out potential contaminating pathogens. We conclude that 16S rDNA sequencing has diagnostic value for patients receiving antibiotic treatment. We regard the method as a complement to culturing when the cultures are negative. Multiple samples per patient should be analysed to determine the clinical significance of positive findings.

## Introduction

Osteoarticular infections present a range of diagnostic difficulties. One of the most challenging aspects of managing these conditions is the final determination of the causative agent(s) [1]. Often, administration of antimicrobials prior to the acquisition of culture specimens jeopardizes the chances of making a microbiological diagnosis via traditional culture-based methods. This study aimed to fill one of the diagnostic knowledge gaps by studying patients with bone and joint infections who had received antimicrobials prior to sampling. In this group, direct DNA sequencing of the sample has a suggested utility [2, 3].

The secondary aim was to assess whether analysing multiple biopsies from each patient had a role in differentiating infection from skin contamination. At present, repeat sampling is standard practice in the culture-based diagnosis of bone and joint infections [4]. To the best of our knowledge, this has not been clearly evaluated with 16S rDNA sequencing techniques. An advantage of this study is the fact that the patients were recruited from routine clinical practice, and the findings can therefore be extrapolated back into the clinical setting.

## Main text

### Methods

Over a period of 4 months, pieces of bone and joint tissue measuring at least 1 mm^3^ were collected from all biopsy specimens arriving for cultures at Karolinska University Laboratory, Stockholm, Sweden. These samples were frozen. The remaining biopsy tissue was cultured for 6 days in Fastidious Anaerobe Broth (FAB) (LAB M Limited, Topley House, UK). Positive broth cultures were subcultured for at least 2 days on blood agar (aerobic and anaerobic), haematin agar (5% CO2) and CLED agar. All samples were collected perioperatively. Referrals of the frozen biopsies were identified, biopsies from Karolinska University Hospital in Solna and Danderyd Hospital (with a total inpatient capacity of 900 and 425 beds at the time of the study, respectively) were chosen, and the respective patient charts were reviewed. Patients who met the criteria for PJI and FRI [4, 5] or suffering from postoperative septic arthritis, defined as a case of septic arthritis after a surgical procedure such as arthroscopy or cruciate ligament reconstruction, who were receiving preoperative antibiotic treatment were included in the study. Preoperative antibiotic treatment was defined as any antibiotic given within 14 days of the surgical intervention to obtain the biopsies. Antibiotic prophylaxis was included in this definition.

Sample tissue was subjected to automated total DNA extraction using the DNA mini protocol for the Biorobot M48 (Qiagen, Hilden, Germany) according to the manufacturer’s instructions. Approximately 100 mg of tissue was subjected to an overnight incubation in Proteinase K and G2 buffer, followed by automated extraction using the Biorobot. Following DNA extraction, an ~ 460-bp region of the 16S rRNA gene was amplified by real-time PCR followed by a chemical purification protocol (ExoProStar) and standard dideoxy nucleotide sequencing based on the Big Dye^®^ Terminator v.3.1 Cycle Sequencing Kit and the Big Dye^®^ XTerminator™ Purification kit. The obtained labelled sequences were separated by capillary electrophoresis technology in an ABI 3100 Genetic Analyser (Applied Biosystems, Foster City, CA, USA) [6].

The DNA sequences were analysed by Seqscape software (Applied Biosystems, Foster City, CA, USA), and nucleotide BLAST database searches were performed for bacterial identification. When mixed chromatograms were encountered, the analysis was carried out with the RipSeq Mixed web application iSentio AS (Bergen, Norway). The culture results were blind to the person analysing the sequencing results. Equivalently sequencing results were blind to the person analysing the culture results. Two patients with two biopsies indicating non-infected tissue but matching primary orthopaedic interventions were used as negative controls in addition to the internal negative controls in the assay (sterile water). The general recommendation in Sweden for PJI and FRI is to culture five separate synovial or bone biopsies in each case of infection and to label the bacterium as the aetiology of the infection if it is present in 3/5 biopsies. However, since it is difficult to sample smaller infected orthopaedic implants five times, we expected to identify multiple cases with less than five biopsy specimens per case. Hence, in our study, a bacterial species was considered an aetiological agent in the infectious episode when it was present in at least half of the biopsies. Likely, environmental contaminants were excluded from the analyses; mainly bacterial species often found in background control samples, such as *Sphingmononas*, *Ralstonia*, and *Lysobacterium*.

## Results

**Fig. 1:**
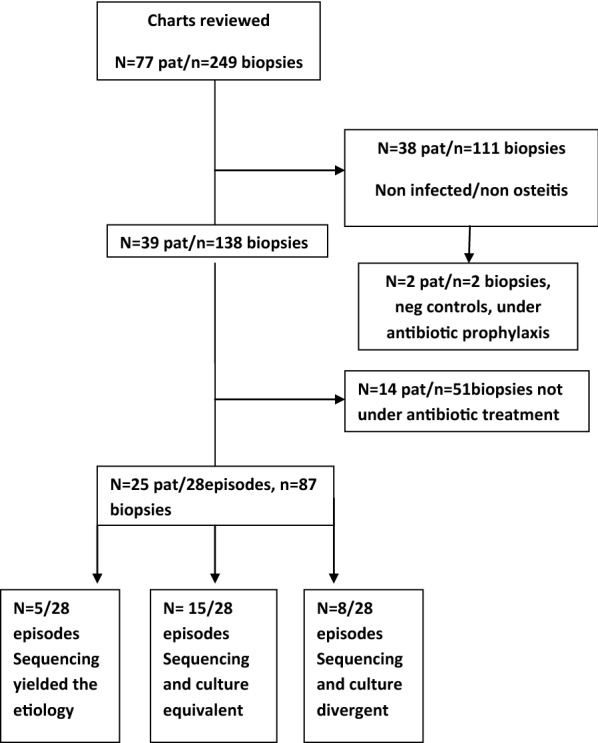
Flowchart of study inclusion

In total, 249 biopsies from 77 patients were collected over the 4-month period of study enrolment. Twenty-five patients with 87 biopsies met the inclusion criteria. Of the patients, 68 percent (17/25) were male and 32% (8/25) female. A flow chart of the study inclusion/exclusion process is displayed in Fig. 1. The average age was 54 years. Three patients were sampled twice: one patient on days 0 and 8, the second patient on days 0 and 11, and the third patient on days 0 and 14. We decided to consider these three patients as having had two episodes of infection, making the total number of patients 25 with 28 infectious episodes. Twenty-nine percent (8/28) of the episodes were PJI, 57% (16/28) were FRI and 14% (4/28) were postoperative septic arthritis. On average, sequencing was performed on three biopsies per infectious episode. The corresponding average for cultures was four biopsies. The discrepancy was due to some biopsies being too small to perform safe division/separation. In this case, the cultures were always prioritized.

The sequencing and culture results are displayed in Table 1 and Fig. 1. In 5/28 episodes, sequencing of several samples identified likely environmental contaminants or provided a more accurate conclusion on the aetiology (Table 2). Culturing of multiple samples provided a more accurate aetiology or ruled out contaminants in 6/28 episodes. For patient #4, multi-sample sequencing helped rule out a pathogen as the causative agent of the infection and instead labelled the pathogen as a contaminant. In this case, *Klebsiella* spp. was found in the sequencing results of only one biopsy sample. The other five biopsies showed other findings. The corresponding cultures from the same episode were either negative or showed a variety of coagulase-negative staphylococci, also indicative of contamination. There were also two cases in which the presence of *Bacillus* spp. was interpreted differently due to multi-sample sequencing. In patient #5, *Bacillus *spp. was labelled as a contaminant when identified in 1/3 of the biopsies. The corresponding cultures were negative. In patient #7, however, *Bacillus *spp. was labelled as responsible for the infection when present in 4/4 sequenced biopsies. In the same patient, the corresponding cultures were negative. In 5/28 episodes, sequencing provided the aetiology of the infection when cultures failed. Here, sequencing identified the responsible bacteria in 4/28 culture-negative episodes, and in 1/28 episodes, sequencing gave the full polymicrobial aetiology. In one episode, a highly significant microbial finding, *Staphylococcus aureus*, was identified with sequencing but not with cultures. In four episodes, culture failed to identify *Bacillus *spp. and *Enterobacterales* (Patients #7, 9, 22, and 25). The culture and sequencing results agreed in 15/28 episodes. In 8/28 episodes, sequencing and cultures produced divergent results. The negative control specimens showed no detectable ribosomal DNA, and the internal control assays were also negative.

**Table 1:** Patient characteristics and results of culture/sequencing

Case no.	Infection	Antibiotic treatment	Days on antibiotic treatment when sampled	No. biopsies for sequencing/cultured	16S ribosomal DNA findings^a,b^	Culture findings^a^
16S rDNA sequencing yielded the full etiology of the infection opposed to culture						
7 = 9	PJI hip	Cloxacillin	11	4/6	*Staphylococcus aureus* + *Bacillus* spp.^d^	Culture negative^c^
9 = 7	PJI hip	Cloxacillin	19	4/4	*S. aureus* + *Bacillus* spp.	*S. aureus*^e^
21	PJI knee	Cloxacillin + Rifampicin	10	3/7	*S. aureus*	Culture negative
22 = 19	FRI upper limb	Clindamycin + Trimethoprim-sulfamethoxazole	12	4/4	*Enterobacterales*	Culture negative
25	FRI pelvis	Linezolid	60	2/2	*Enterobacterales*	Culture negative
16S rDNA sequencing equal to conventional culture in deciding the causative agent of the infection						
1	FRI hip	Imipenem	7	2/2	*Escherichia. coli*	*E. coli*
5	Postop arthritis	Cloxacillin	4	3/3	Not detected^f^	Culture negative
4	Postop arthritis	Cloxacillin	1	6/9	Not detected^g^	Culture negative^g^
2	Postop arthritis	Cefotaxime	8	1/1	*E. coli*	*E. coli*
6	FRI hip	Isoxazolyl-penicillin	7	− 2/2	Not detected	Culture negative
12	FRI lower limb	Clindamycin	1	1/1	*S. aureus*	*S. aureus*
13	PJI hip	Cefuroxime	3	7/7	*S. aureus*	*S. aureus*
14	FRI lower limb	Cefotaxime	3	1/1	Not detected	Culture negative
15	FRI hip	Cefotaxime + Clindamycin	2	5/5	*S. aureus*	*S. aureus*
16	PJI hip	Cloxacillin + Rifampicin	18	3/3	*Pseudomonas aeruginosa*	*P. aeruginosa*
17	Postop arthritis	Ciprofloxacin	9	1/6	Not detected	Culture negative
19 = 22	FRI upper limb	Cloxacillin + Penicillin G	3	2/2	*Bacillus cereus*	B*. cereus*
23	FRI lower limb	Cloxacillin + Clindamycin	1	2/3	Not detected	Culture negative
28	FRI upper limb	Prophylaxis only—Clindamycin	0	3/3	*S. aureus*	S. *aureus*
27	PJI hip	Ceftriaxone	3	6/6	*Streptococcus dysgalactiae*	*S. dysgalactiae*
Different results when comparing conventional culture and 16S rDNA sequencing						
18	FRI upper limb	Isoxazolylpenicillin	28	1/1	Not detected	*S. aureus*
20 = 24	FRI lower limb	Isoxazolylpenicillin + Ciprofloxacin	13	1/5	*Enterobacterales* + *Enterococcus* spp. + *Anaerococcus* spp.	*Citrobacter freundii* + *Enterococcus faecalis* + *Enterococcus faecium*
24 = 20	FRI lower limb	Imipenem	60	4/4	*Enterococcus* spp.^h^	*E. faecium*
26	FRI pelvis	Imipenem + Linezolid	13	6/6	Not detected	CoNS^i^
8	PJI hip	Cefuroxime	3	7/7	*Proteus mirabilis*	*P. mirabilis* + *Corynebacterium* spp.
3	FRI spine	Cloxacillin	5	2/2	*S. aureus*	*S. aureus* + *Cutibacterium acnes*
10	PJI knee	Clindamycin	5		*Acinetobacter* spp. + *E. faecalis*	*Acinetobacter* spp. + *E. faecalis* + CoNS
11	FRI lower limb	Cloxacillin + Penicillin G	9	2/2	Not detected	CoNS

*PJI* prosthetic joint infection; *FRI* fracture related infection; *CoNS* coagulase negative staphylococci; *rDNA* ribosomal DNA; *S. aureus Staphylococcus aureus*, *B. cereus Bacillus cereus*; *S. dysgalactiae Streptococcus dysgalactiae*; *E. coli Escherichia coli*; *Spp.* species; *P. aeruginosa Pseudomonas aeruginosa*; *faecalis Enterococcus faecalis*, *Enterococcus faecium*

^a^Relevant finding defined as presence in at least 50% of the samples in the case of multiple samples

^b^Environmental bacteria excluded from the analysis

^c^Later biopsies showed *S. aureus* in all biopsies and *B. cereus* in only one biopsy

^d^*B.* c*ereus* in 3/3 biopsies

^e^*B. cereus* in 1/4 biopsies

^f^*S. aureus* in 1/3 biopsies. A clinician would likely regard this finding as significant despite diagnostic criteria. *Bacillus* spp. in 1/3 biopsies

^g^Environmental contaminants excluded through analysing several biopsies

^h^Listed as discrepant since sequencing failed to identify enterococcal species, a disadvantage concerning choice of antibiotic

^i^Multiple strains of CoNS, suspected contamination

**Table 2:** Detailed culture and sequencing results in episodes where multiple-sample sequencing added value in final determination of causative agents

Patient no	Type of infection		Biopsy 1	Biopsy 2	Biopsy 3	Biopsy 5	Biopsy 6	Biopsy 7
4	FRI	Sequencing	*Klebsiella* spp.	*Streptocuccus australis*	*Ralstonias* spp.	*Alcalimonas* spp.	*Flavobacteriaceae*	CoNS strain 1
		Culture	CoNS strain 1	CoNS strain 2	Culture negative	Culture negative	Culture negative	CoNS strain 2
5	Postoperative arthritis^a^	Sequencing	*S. aureus*	Not detected	*Bacillus* spp.			
		Culture	Culture negative	Culture negative	Culture negative			
11	FRI	Sequencing	*Burkholderia cepacia*	*Bacillus* spp.				
		Culture	CoNS	CoNS				
7	PJI	Sequencing	*Bacilllus* spp.	*S. aureus* + *Bacillus* spp.	*S. aureus* + *Bacillus* spp.	*S. aureus* + *Bacillus* spp.		
		Culture	Culture negative	Culture negative	Culture negative	Culture negative		
25	FRI	Sequencing	*Klebsiella* spp.	*Micrococcus luteus*				
		Culture	Culture negative	Culture negative				

*S. aureus* *Staphylococcus aureus*; *Spp. *Species; *CoNs *coagulase negative staphylococci; *FRI *fracture related infection; *PJI *prosthetic joint infection

^a^After cruciate ligament reconstruction

## Discussion

Analysing several biopsies with sequencing in each case of infection was of major clinical importance in selected cases. In contrast, the same and more universally accepted modus operandi to culture several biopsies per infectious episode was of value compared to sequencing in only one additional case (6/28 vs. 5/28 episodes). We argue that several biopsies should be analysed per patient with osteoarticular infections if the patient has received antibiotic therapy. Repeat sampling mitigates the risk of contamination regardless of whether culture-based or sequencing methods are used. This approach will increase costs but logically, cost should not be the only aspect considered, as with cultures. The question of how many biopsies to analyse per patient remains unanswered. Patel et al. concluded that four samples allow for an accurate diagnosis using culture-based methods [7]. Our sample numbers are not large enough to either refute or substantiate this statement.

In this cohort of patients who received antimicrobials prior to sampling, we estimate that sequencing provided the aetiology of the infection in 5/28 episodes when cultures failed or did not show the complete polymicrobial aetiology (as in case #9). Similar results were achieved in a study by Bemer et al. [8], where sequencing yielded a bacterial diagnosis of PJI in 50% of culture-negative cases in patients receiving antibiotic treatment. Additionally, Parvizi et al. concluded that next-generation sequencing identified aetiologies in 9/11 culture-negative cases [2]. Based on our results and the results by Bemer and Parvizi, we regard sequencing as an important complement to cultures that should be utilized in cases where false-negative cultures are suspected. One possible approach would be to preserve peri-operative biopsy material and perform sequencing only when there is a negative culture. This is logistically challenging but given the unacceptably poor outcomes of patients with culture-negative PJI [9], this approach may be of critical importance.

Among the cases where the sequencing and culture results differed, the majority were of cultures detecting more potential contaminants. These were mainly bacterial species of low virulence. This finding has also been seen in other studies [10]. Since foreign material was present in all episodes that led to differing sequencing and culture results in our study, it can be hard to exclude or verify whether these low virulent bacteria are causing the infection. This is well known since low virulent bacteria such as *Cutibacterium acnes* and CoNS have the ability to infect foreign material differently than native tissue [11]. This further stresses the importance of diagnostic algorithms to help clinicians judge such results. Importantly, culture media have the potential to enrich environmental contamination, whereas no such media is used when performing sequencing. Other differences in culture and sequencing results were attributable to the 16S rDNA method being unable to identify some staphylococci, *Enterobacterales* and enterococci to down to the species level [12]. This problem has been partly alleviated by the introduction of next-generation sequencing, but species identification remains a challenge.

In our study, the sequencing of multiple biopsies was of value in identifying bacterial aetiologies in selected cases. Conversely, there were also cases in which cultures added more value to the diagnosis and subsequent antibiotic therapy. Unfortunately, the sequencing technique used cannot predict antimicrobial susceptibility. In most cases, the 16S rDNA sequencing and culture results agreed. It is noteworthy that all of these patients had received antibiotics, but the cultures were nevertheless positive. Based on our results, we recommend that clinicians use sequencing techniques as a diagnostic method for osteoarticular infections as a complement to conventional culturing, mainly in culture-negative cases. We also recommend analysing multiple samples with both cultures and sequencing.

## Limitations

The small number of patients in our study, which also included patients with a variety of different types of bone and joint infections, precluded statistical analyses. For these reasons, the results are presented in a descriptive manner only.

## Data Availability

The datasets generated during and/or analysed during the current study are available from the corresponding author on request.

## Abbreviations

16S rDNA sequencing: 16S ribosomal DNA sequencing
PJI: Prosthetic joint infection
FRI: Fracture-related infection
CoNS: Coagulase-negative staphylococci
